# Supplementary material for: Temperature Dependence of the Beating Frequency of hiPSC-CMs Using a MEMS Force Sensor
Source: Sensors (Basel). 2023 Mar 23;23(7):3370. doi: 10.3390/s23073370 (PMC10098744; doi:10.3390/s23073370)
Supplement: Supplementary file 1 [file sensors-23-03370-s001.zip › sensors-2290341-supplementary.pdf]

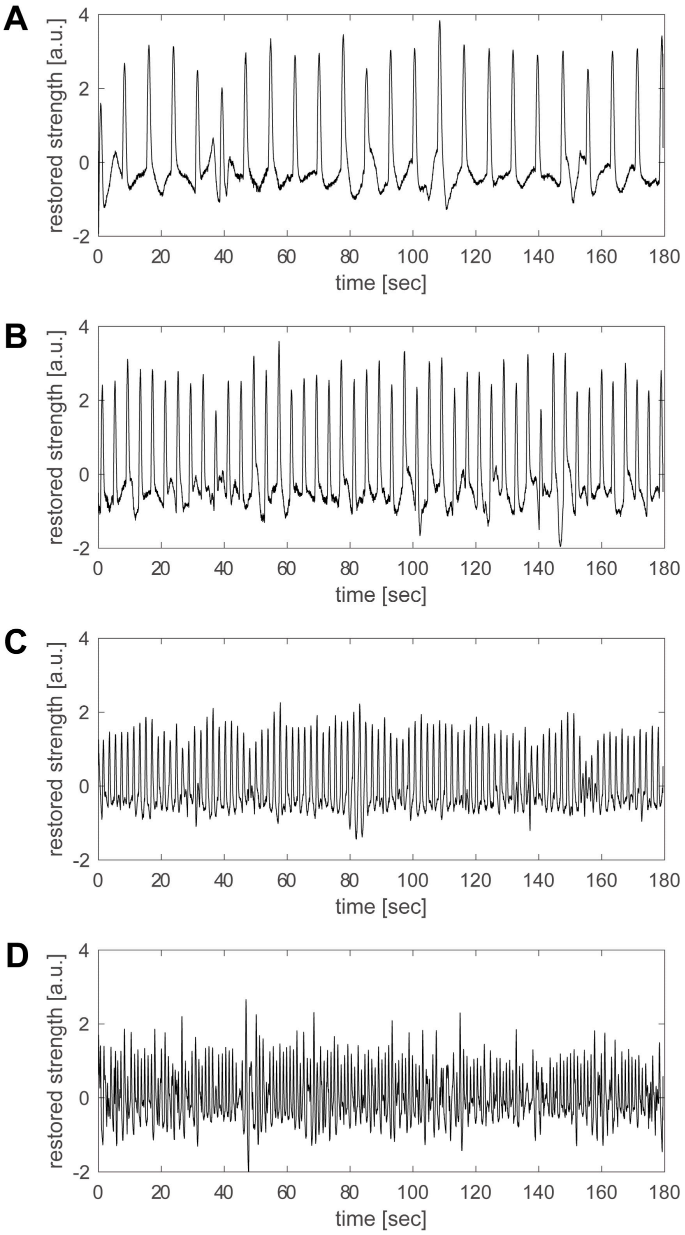

Figure S1. Beating data of hiPSC-CMs group A at various temperatures. These data correspond to Figure 7 in the manuscript. Each graph is the signal at the temperatures shown below. (A) 22℃ (B) 24℃ (C) 28℃ (D) 32℃.

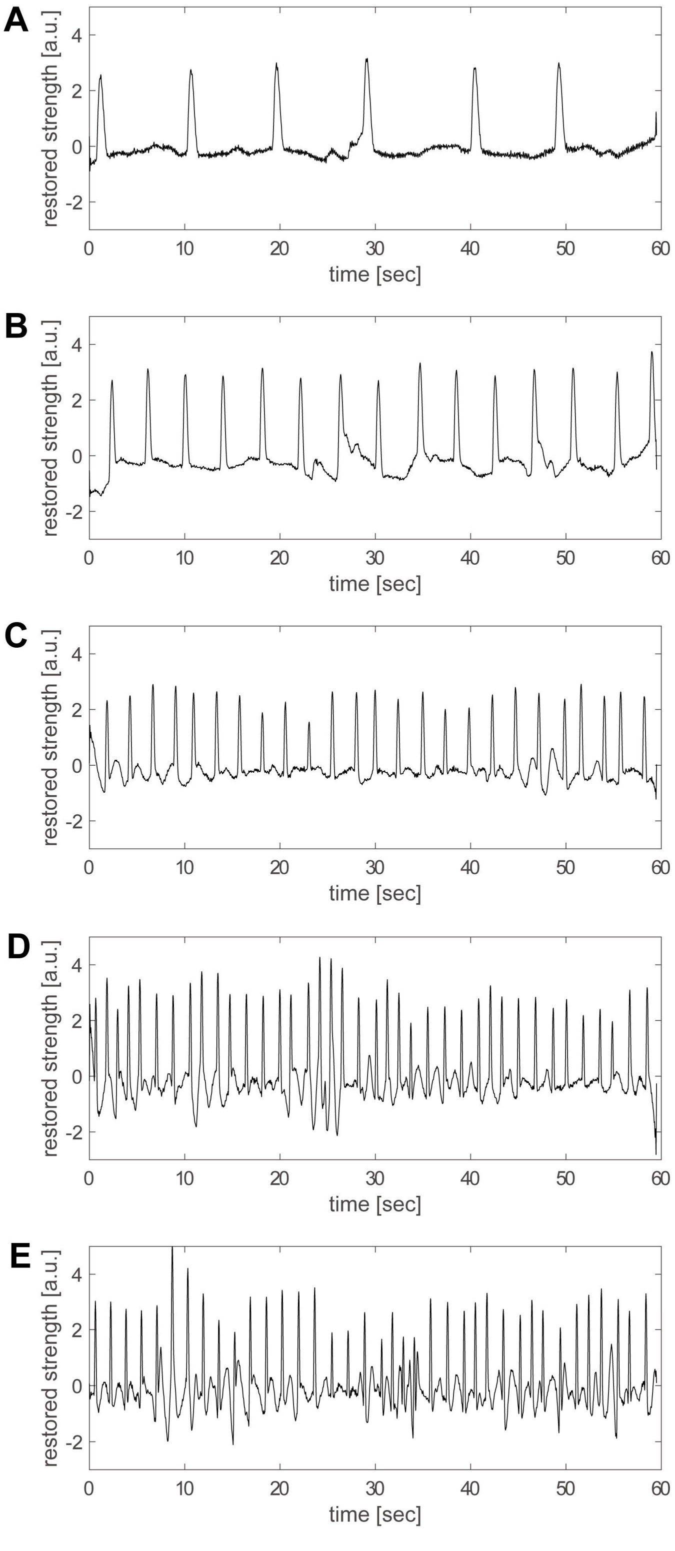

Figure S2. Beating data of hiPSC-CMs group B at various temperatures. These data correspond to Figure 7 in the manuscript. Each graph is the signal at the temperatures shown below. (A) 22°C (B) 24°C (C) 28°C (D) 32°C (E) 36°C.

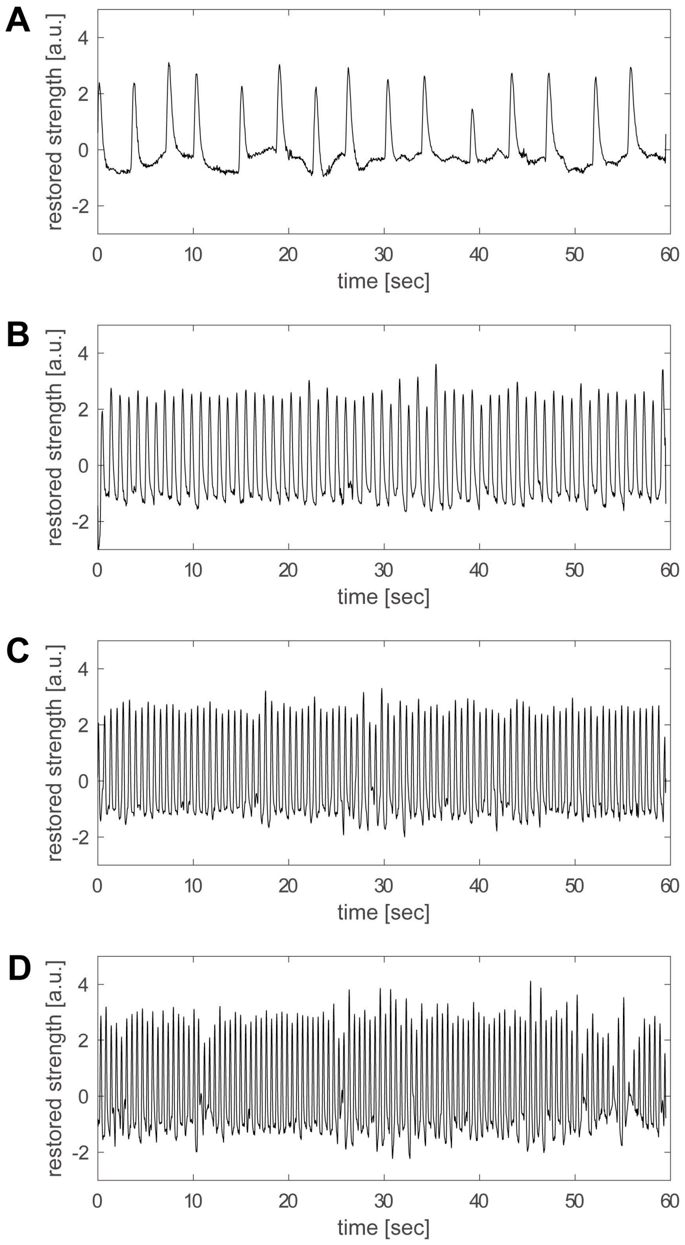

Figure S3. Beating data of hiPSC-CMs group C at various temperatures. These data correspond to Figure 7 in the manuscript. Each graph is the signal at the temperatures shown below. (A) 22℃ (B) 24℃ (C) 28℃ (D) 32℃.

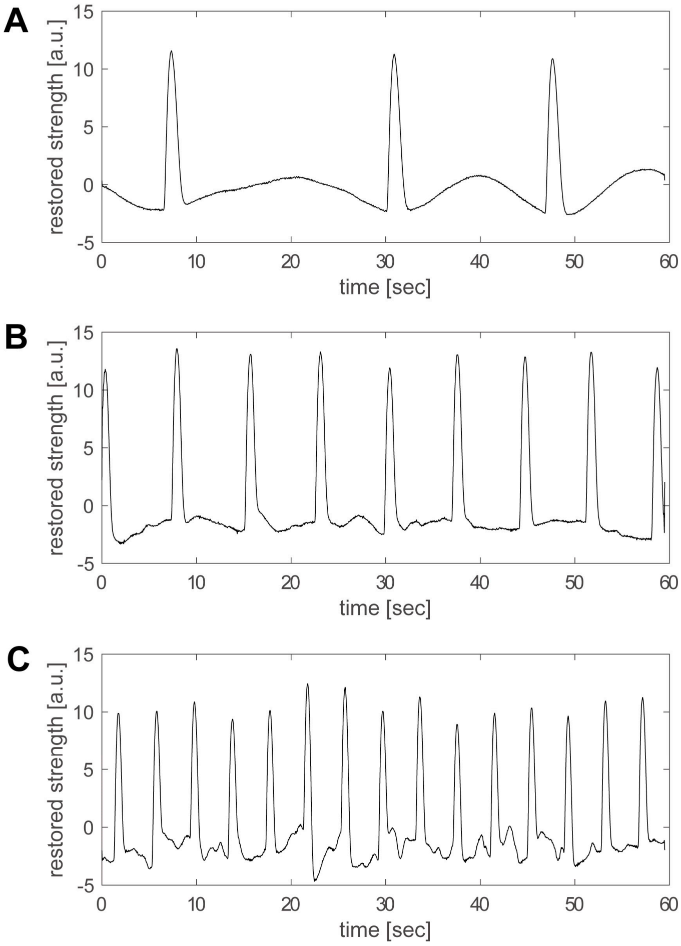

Figure S4. Beating data of hiPSC-CMs group E at various temperatures. These data correspond to Figure 7 in the manuscript. Each graph is the signal at the temperatures shown below. (A) 22℃ (B) 24℃ (C) 28℃.

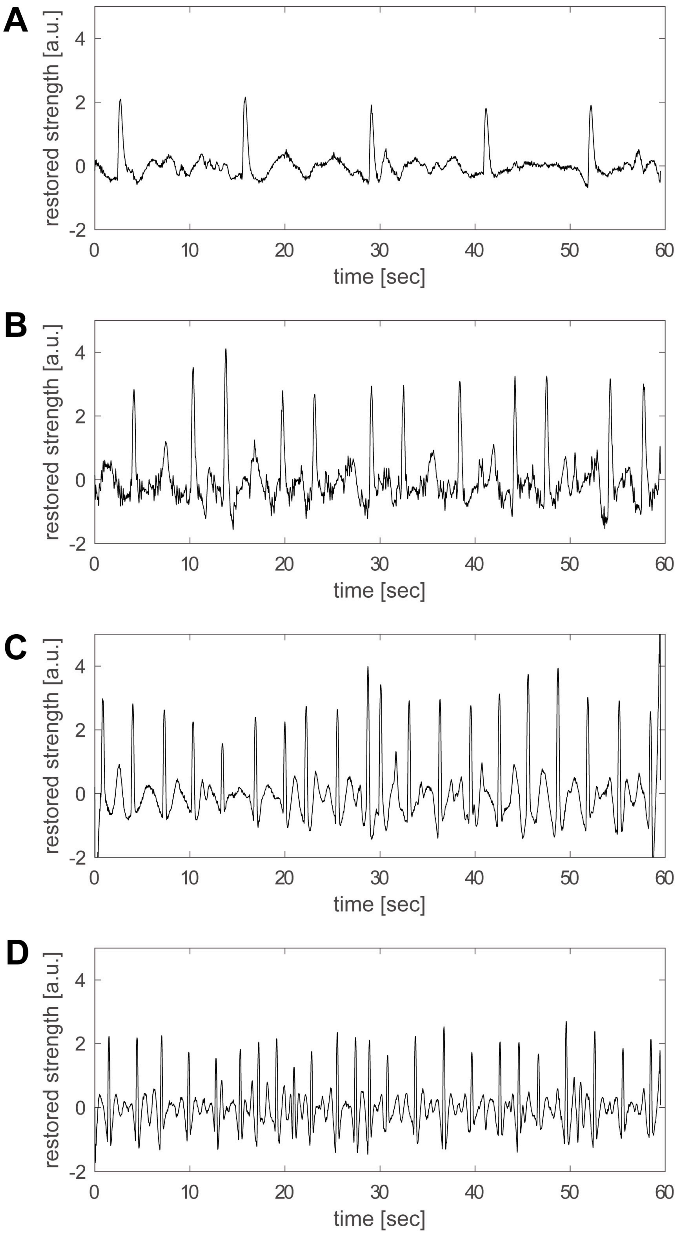

Figure S5. Beating data of hiPSC-CMs group F at various temperatures. These data correspond to Figure 7 in the manuscript. Each graph is the signal at the temperatures shown below. (A) 22°C (B) 24°C (C) 28°C (D) 32°C.

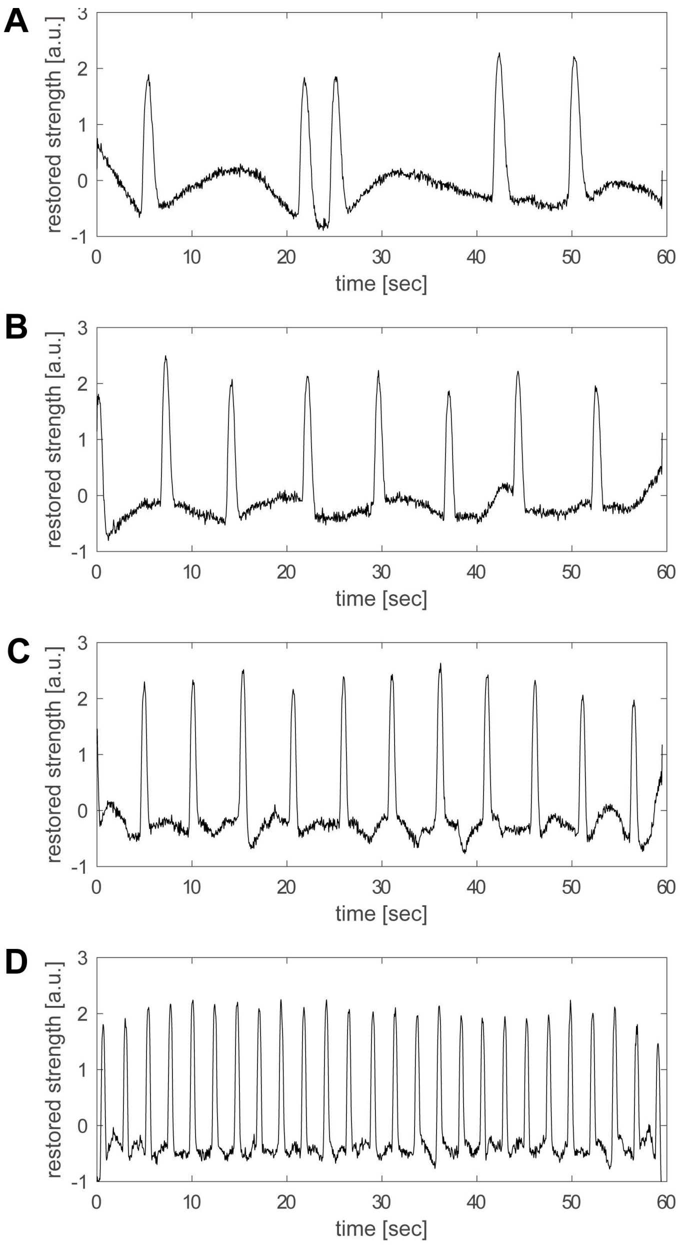

Figure S6. Beating data of hiPSC-CMs group G at various temperatures. These data correspond to Figure 7 in the manuscript. Each graph is the signal at the temperatures shown below. (A) 22°C (B) 24°C (C) 28°C (D) 32°C.

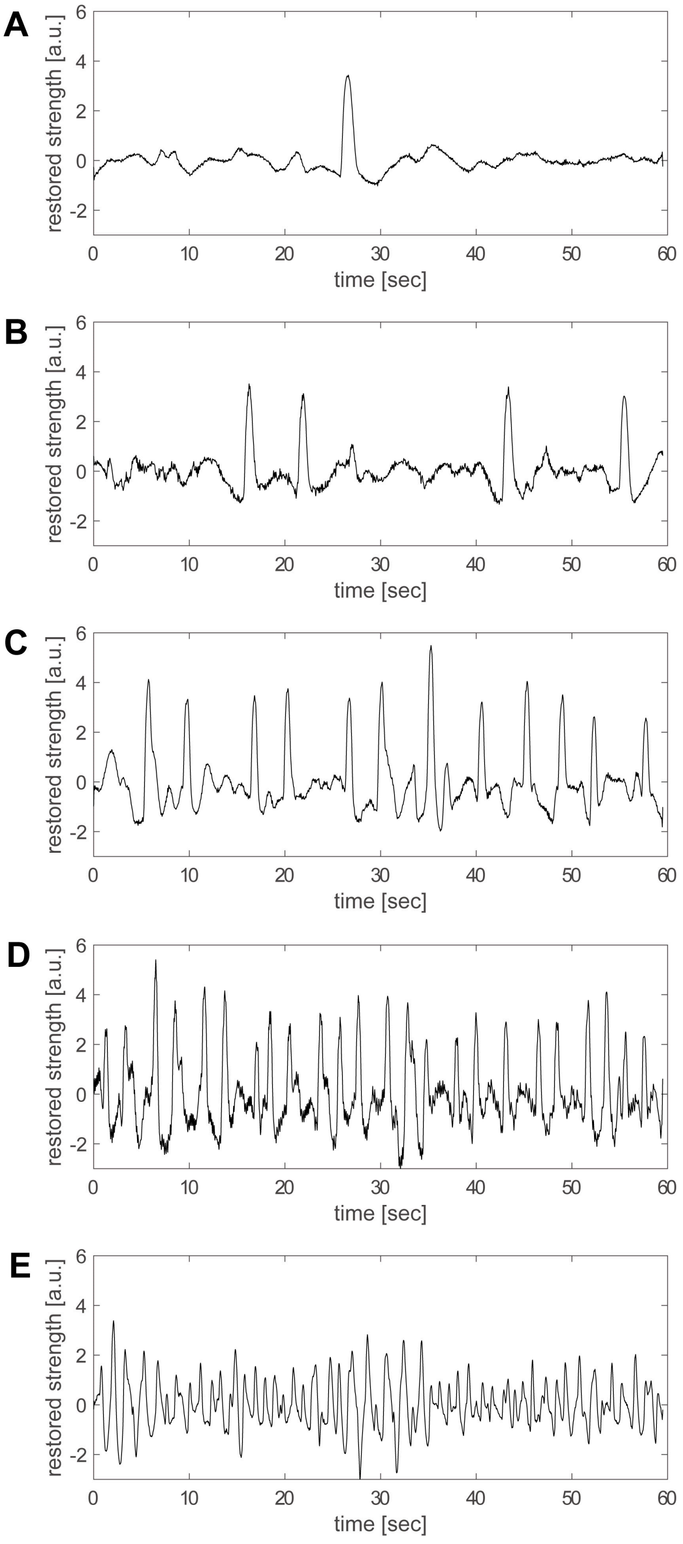

Figure S7. Beating data of hiPSC-CMs group H at various temperatures. These data correspond to Figure 7 in the manuscript. Each graph is the signal at the temperatures shown below. (A) 22℃ (B) 24℃ (C) 28℃ (D) 32℃ (E) 36℃.

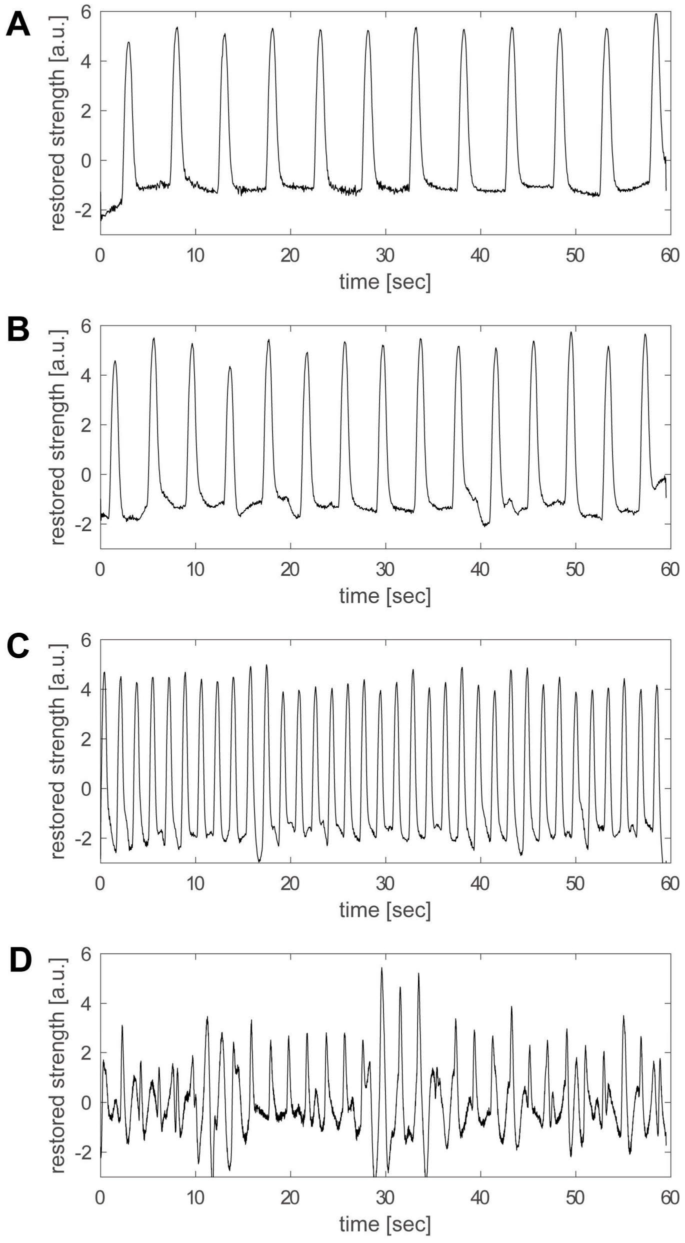

Figure S8. Beating data of hiPSC-CMs group I at various temperatures. These data correspond to Figure 7 in the manuscript. Each graph is the signal at the temperatures shown below. (A) 22℃ (B) 24℃ (C) 28℃ (D) 32℃.

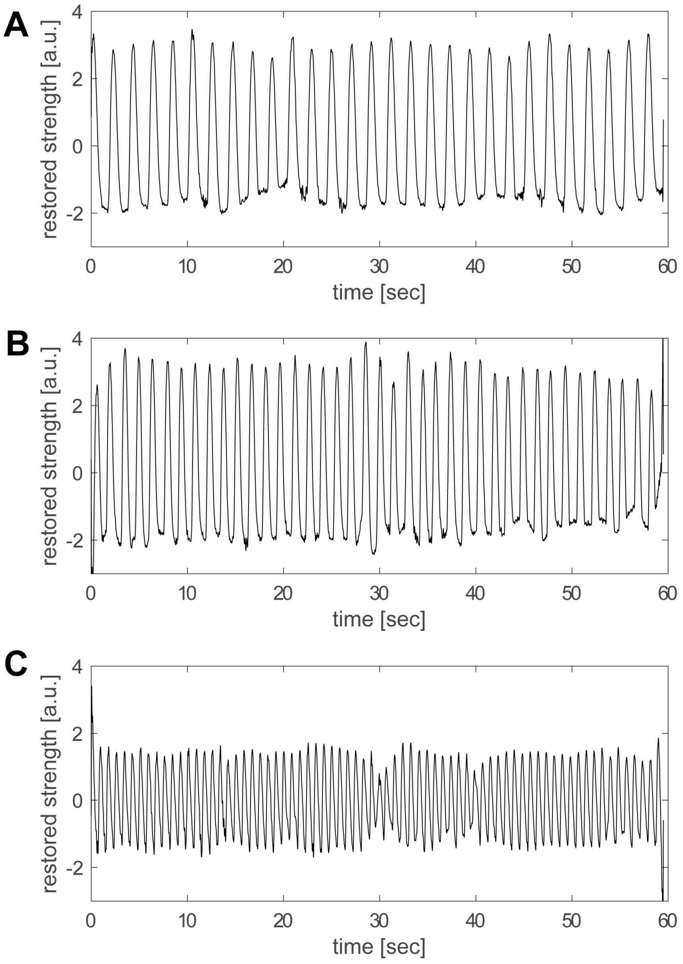

Figure S9. Beating data of hiPSC-CMs group J at various temperatures. These data correspond to Figure 7 in the manuscript. Each graph is the signal at the temperatures shown below. (A) 22℃ (B) 24℃ (C) 28℃.

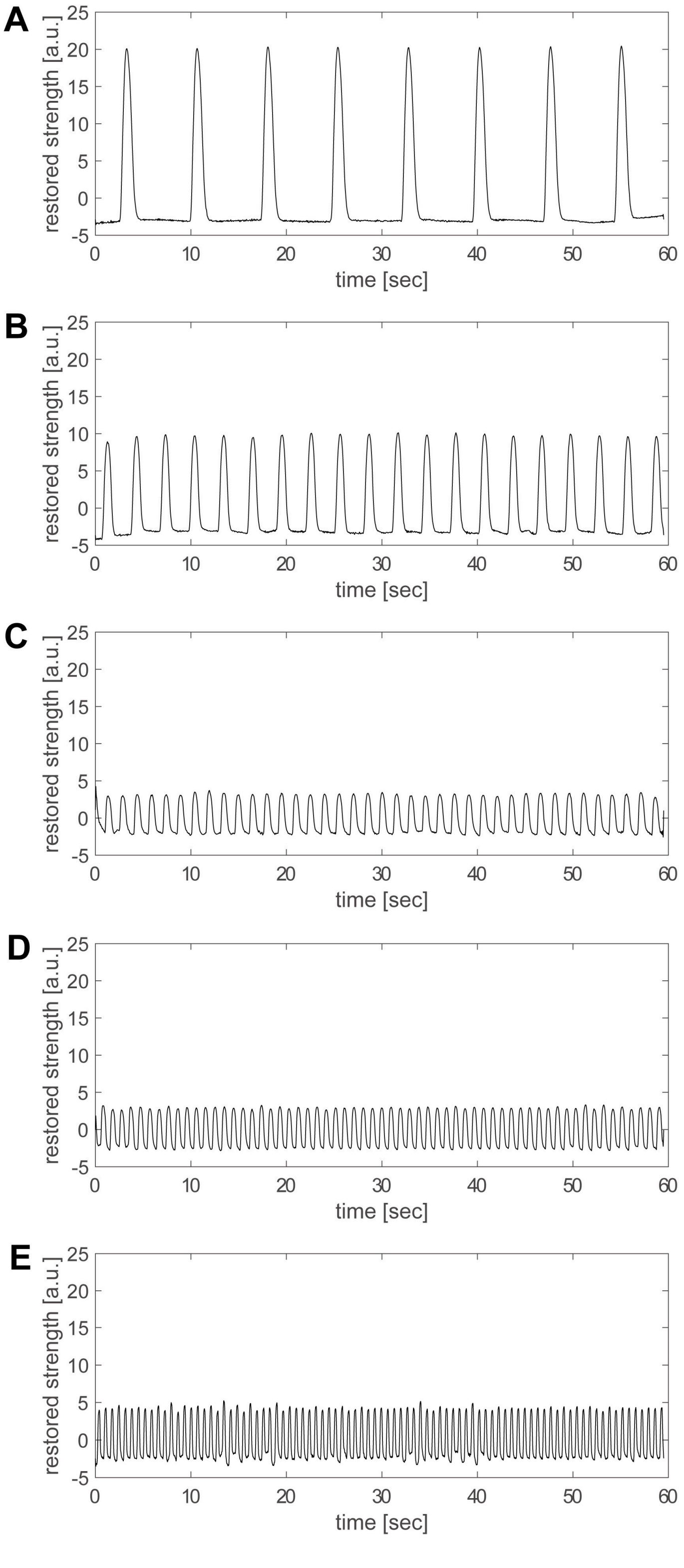

Figure S10. Beating data of hiPSC-CMs group K at various temperatures. These data correspond to Figure 7 in the manuscript. Each graph is the signal at the temperatures shown below. (A) 22°C (B) 24°C (C) 28°C (D) 32°C (E) 36°C.
